# Professional Male Beach Handball Players Performance Profile

**DOI:** 10.3390/nu14224839

**Published:** 2022-11-15

**Authors:** Alejandro Martínez-Rodríguez, Javier Sánchez-Sánchez, María Martínez-Olcina, Manuel Vicente-Martínez, Rodrigo Yáñez-Sepúlveda, Guillermo Cortés-Roco, Juan Antonio Vázquez-Diz, Juan Antonio Sánchez-Sáez

**Affiliations:** 1 Department of Analytical Chemistry, Nutrition and Food Science, University of Alicante, 03690 Alicante, Spain; 2 Alicante Institute for Health and Biomedical Research (ISABIAL), 03010 Alicante, Spain; 3 School of Sport and Science, European University of Madrid, 28670 Madrid, Spain; 4 Faculty of Health Science, Miguel de Cervantes European University, 47012 Valladolid, Spain; 5 Faculty of Education and Social Sciences, Universidad Andres Bello, Vina del Mar 2520000, Chile; 6 Escuela de Educación, Pedagogía en Educación Física, Entrenador Deportivo, Universidad Viña del Mar, Vina del Mar 2520000, Chile; 7 Departamento Educación Física y Deporte, Universidad de Sevilla (E. U. Osuna), 41640 Osuna, Spain; 8 Grupo de Investigación GDOT-Gestión Deportiva, Ocio y Tecnología, Faculty of Sport, Catholic University of Murcia, 30107 Murcia, Spain

**Keywords:** performance test, male sports performance, sand sports, team sports

## Abstract

Beach handball is a sport that has seen an increase in the number of players in the last decade. The aim of the study was to evaluate the basic measures of body composition, maturation, performance outcomes and the adherence to the Mediterranean diet of professional beach handball players, as a function of category (junior vs. senior) and playing positions. Thirty-six professional beach handball players participated in the study: 18 seniors (age: 25.0 ± 5.19) and 18 juniors (age: 16.7 ± 0.46); Each player’s body composition and different sports performance variables were analysed using the CMJ test, the Abalakov test, the Yo-Yo Test IR1 and the handgrip test. The Mediterranean diet Adherence was analysed using the KIDMED questionnaire. Significant differences are observed for all performance variables, with the values of senior players being higher in all cases (*p* < 0.005). Moreover, the adherence to the Mediterranean diet is moderate. Positive correlations were observed between the CMJ and weight (*p* = 0.012) and the CMJ and the BMI (*p* = 0.003). The same was observed for the Abalakov test, with *p* = 0.004 for weight and *p* = 0.001 for the BMI. Regarding the dynamometry, it positively correlates with the height (*p* = 0.002), the sitting height (*p* = 0.008), wingspan (*p* < 0.001) and weight (*p* = 0.011). The opposite occurred with the Yo-Yo Test and the KIDMED variables. Conclusions: One aspect to improve, as a key performance factor, would be the adherence to the Mediterranean diet for both categories. Players with a better adherence, achieved better results in the performance tests.

## 1. Introduction

Beach handball (BH) is a discipline that is derived from indoor handball, which originated in Italy in the early 1990s [1]. However, it is only in the last few years that this sport has really become the focus of attention of researchers. Studies on the characteristics of this sport, such as tactical decision-making [2,3], anthropometric profiles, body composition or bone quality [4,5,6,7], physical and physiological demands [8,9,10,11] and even psychological profiles, levels of competitive anxiety, mood and self-efficacy or eating disorders [12,13] of beach handball players, have been published. The required efforts are of an intermittent nature with game actions between six and 15s long, however depending on the sex of the players, the physical demands required vary [3,4,5,6,7,8,9,10,11,12,13,14]. This type of effort is due to the idiosyncrasy of the sport itself (a two-set match of 10 min with a play-off round, if necessary), so it is vital to know the physical demands of the player during the game [15,16]. In this sport, high-intensity actions predominate, with short recovery periods and constant role changes of the players during the match [17].

To achieve an optimal performance of BH players, they should have their weight and fat percentage within the recommended range for their age group, position, and gender [4,5,6,7]. This is because there are direct relationships between body composition and performance tests [5,18]. The size of the body is of great importance for both attacking and defensive blocking, and it is possible to achieve higher throwing velocities and isometric strength, when size is greater [9,19,20].

The internal load demands caused by the practice of BH (physiological demands) are derived from intermittent efforts [11]. For this reason, it is considered a high-intensity sport, as most of the playing time is spent in the game and the player’s HRMAX is above 80% of [10,11,21,22]. Other characteristics to be considered that directly impact the internal load of the athlete would be the kinematic variables in the male players [20]: (a) distance: 870 ± 217 m (male juniors)/790 ± 205 m (male adults) and (b) maximum speed: 17.3 ± 2.0 km/h) (male juniors)/16.3 ± 2.0 km/h (male adults).

To measure the performance variables in team sports, there are different instruments and validated tests that provide fundamental data to evaluate the physical and physiological profile of the athletes. However, one of the most widespread is the Yo-Yo intermittent recovery test (level) 1 [18,22,23,24], whose only reference applied to BH is found in the research of Martínez-Rodríguez et al. [6]. Other widely used tests are the Abalakov jump test (AJT) and the moving jump (CMJ), which are used to determine fatigue and the lower body power in athletes [20]. In relation to muscle strength, the handgrip test (HT) is used to measure the upper body musculoskeletal strength and muscle fatigue [8,11], applied to BH players. In both cases, the research of Sánchez-Sáez et al. [11] was used as a baseline BH study, that determined that there was no significant reduction in the jumping ability or upper body strength throughout the duration of the game, in beach handball players (*p* > 0.05).

Due to the high physical demands of the sport, the proper selection of food and fluid intake by players is of great importance [25]. Considering the total energy intake, depending on the duration and intensity of the training, as well as the quality of the food itself, it is necessary to achieve and maintain the body weight, to improve the players’ health and results of the training. The dietary pattern that has the most positive effects on health is the Mediterranean diet (MD) [26,27,28,29].

The objectives of the research were to evaluate the basic measurements of the body composition, performance outcomes and adherence to the Mediterranean diet of professional beach handball players, depending on the category (junior vs. senior) and playing position, as well as to study the possible relationships between the variables. As an initial hypothesis, it is expected that senior players will have higher values for height, sitting height, wingspan, weight and performance outcomes, due to their increased professionalization. The positive correlations are hoped to be found between the performance outcomes and the adherence to the MD.

## 2. Materials and Methods

### 2.1. Design of the Study

A cross-sectional, descriptive study was performed to analyse the influence of both age categories and playing positions, on the performance test scores and adherence to the median diet. In addition, the maturation was analysed, in the case of the junior category. The best international players from Spain, in this sport, participated in the research. They represent the BH elite from all over the world. The guidelines of the Declaration of Helsinki and the recommendations of Good Clinical Practice of the EEC (Document 111/3976/88 of July 1990) were considered for all procedures. The Ethics Committee of the University of Alicante (Spain) (UA-2019-04-09) approved the research.

### 2.2. Eligibility Criteria and Participants

The study involved thirty-six professional beach handball players: 18 senior and 18 junior players. The distribution of the sample by playing position were: six goalkeepers, 12 wings, six specialists, five pivots and seven defenders, all of them were part of the Spanish national BH team. The presence of a chronic disease, an injury during the intervention period that impeded the performance of any of the tests, and non-compliance with the informed consent, were the exclusion criteria established. Nevertheless, all participants gave their consent. In the case of the minors, consent was given by their parents or legal guardians. The anonymity of the players was always maintained. The players did not receive any financial compensation for their participation.

### 2.3. Data Collection

#### 2.3.1. Body Composition

The four basic means of the international standards for anthropometric assessment (ISAK) were performed; weight, height, sitting height and wingspan. Weight was assessed using the validated Tanita BC-730F bioimpedance scale (Tanita, Amsterdam, The Netherlands). The height and sitting height were determined, using a mobile anthropometer (Seca 213, SECA Deutschland, Hamburg, Germany), with the participants’ heads held in the Frankfurt horizontal plane position. The wingspan (greatest distance between the points of the middle finger of the right and left hands, expressed in centimetres) was measured, using an anthropometer (Smartmet, Jalisco, Mexico), attached to the wall and parallel to the floor. The body mass index (BMI) was calculated from the weight and height; kg/m^2^.

#### 2.3.2. Performance Test

To measure the lower limb explosive strength, the CMJ and Abalakov tests [20] were performed. Using an infrared contact platform (Optojump^®^, Microgate, Bolzano, Italy), both tests were performed. The players performed each test three times, with a 30 s rest between jumps. Of these three results, the best one was used for the statistical analysis.

During the CMJ, the players were instructed to place their hands on their hips to minimize the lateral and horizontal displacement during execution [30,31], to avoid any influence of arm movements on the vertical jumps. The CMJ involved participants to descend as quickly as possible from an upright position to a self-selected depth, followed immediately by a vertical jump. The participants were instructed to jump as high as possible.

The Abalakov test was performed similarly to the CMJ, except that the upper limbs had the freedom of action [20]. The players started from an upright position and an upward jump was performed by means of a stretch-shortening cycle (i.e., a flexion followed, as quickly as possible, by a leg extension with the free influence of the arms assessing the reflex-elastic-explosive manifestation and the utilization rate of the lower extremities).

The strength of the upper extremities of the body was measured with the Jamar hand-held hydraulic dynamometer. This test is reliable for assessing the musculoskeletal fitness of the upper extremities in athletes [32]. The participants pressed gradually and continuously for at least two seconds, performing the test with the dominant hand, with the arm forming a 90° angle. The test was performed three times for each hand, with an interval of 1 min, and the maximum score for each hand was recorded in kilograms of force (kg). The highest value recorded was retained for the analysis.

Following the procedures described by Krustrup et al. [33], the test known as the Yo-Yo test IR1 was performed. This test required the participants to perform 2 × 20 m of running interspersed with 10s of recovery at progressive speeds, dictated by a pre-recorded audio-graphic signal. Previously, the participants completed a warm-up, based on low intensity running and joint mobility, directed by a sport scientist. The test was stopped when the participant did not reach the first line in time (the objective assessment), twice in a row, or if they felt they would not be able to complete another sprint (subjective assessment). The final score was recorded as the total distance covered. The reliability of the Yo-Yo test IR1 test was demonstrated by an inter-test coefficient of variation (CV) of 3.6% and an intra-class correlation coefficient (ICC) of 0.94 [22].

#### 2.3.3. Mediterranean Diet Adherence (KIDMED Questionnaire)

To determine the adherence to the Mediterranean diet, the KIDMED questionnaire was used [34,35]. There were 16 items in this questionnaire; there were twelve items representing a positive score for the adherence to the Mediterranean diet and four items representing a negative score. A positive response to a question implying the increased adherence, is the equivalent of +1 point. A positive answer to a question that means a low adherence to the diet is worth −1 point. No score is given for negative answers (value 0). All scores are summed up in the KIDMED index, which ranges from 0 to 12 points. Depending on the result obtained, the adherence to the Mediterranean diet is classified as low (very poor-quality diet, 0–3), medium (need to improve the diet, 4–7) or high (adequate adherence to the Mediterranean diet, 8–12) [36].

### 2.4. Statistical Analysis

The Jamovi statistical software (version 1.6.15, The JAMOVI Project, Sydney, Australia) was used for the data analysis. The descriptive data (mean and standard deviation) were calculated. Kolmogorov–Smirnov test was performed to evaluate the normality of the descriptive statistics. To evaluate the normality of the data, Levene’s test was used. To contrast the differences in the baseline measurements, according to the group, the *t* test was used, with the age and BMI (kg/m^2^). To contrast the differences in the variables of the adherence and compliance to the Mediterranean diet between the different categories (junior vs. senior) and playing position, a covariance analysis (ANCOVA) with the Bonferroni adjustment was applied, adjusting for the effect of the BMI. The effect size was also calculated, using a partial eta-squared (ηp2), considering <0.25, 0.26–0.63 and >0.63 as small, medium and large effect sizes, respectively [37]. In the case of the analysis according to the playing position, a partial omega squared (ω^2^) was also calculated, since the sample of each of the groups is smaller (0.01–0.05: small effect, 0.06–0.13: moderate effect, >0.14: large effect) [38].

## 3. Results

Thirty-six male BH players from the Spanish national team participated in the study. Age, basic anthropometric measurements, and BMI, both for the total sample and by age category (junior vs. senior), are shown in Table 1. Between both categories, there were differences in the variables age, height, sitting height, wingspan and weight. In all cases, the variables of the seniors were superior.

As for the performance variables, the adherence to the MD and the maturation are presented in Table 2. Significant differences are observed for all performance variables, with the values of the senior players being higher in all cases (*p* < 0.005). Regarding the total KIDMED score, there are no significant differences. In fact, the scores are similar. However, the results obtained in both cases, as for the total sample, indicate that the adherence to the Mediterranean diet is moderate, and therefore needs to be improved.

Following the performance of an analysis of variance, adjusted for the BMI, no significant differences were observed for any of the variables, as a function of the playing position (Table 3). The same applies to the post hoc analyses.

The correlations between the different variables of the total sample are shown in Table 4. To highlight the correlations between some of the performance results and the anthropometric variables, positive correlations were observed between the CMJ and weight (*p* = 0.012), the CMJ and the BMI (*p* = 0.003), therefore, the greater the weight and BMI, the greater the jump height (cm). The same is true for the Abalakov test, with *p* = 0.004 for the weight and *p* = 0.001 for the BMI. As for the dynamometry, i.e., the upper body strength, it is positively correlated with the height (*p* = 0.002), the sitting height (*p* = 0.008), the wingspan (*p* < 0.001) and the weight (*p* = 0.011). The opposite occurred with the Yo-Yo test and KIDMED variables.

Negative correlations are observed with the weight (*p* = 0.003 and *p* = 0.014 for the Yo-Yo test and KIDMED, respectively) and with the BMI (*p* < 0.001 and 0.011). Therefore, for a higher weight and BMI, the results of the Yo-Yo test are lower (the distance covered is shorter) and the adherence to the Mediterranean diet is worse. The variables of the KIDMED and the Yo-Yo test also correlate positively with each other, therefore, those players who have a better adherence to the Mediterranean diet, covered a greater distance (*p* = 0.002) and obtained higher VO_2_ max. values (*p* = 0.002). Regarding the performance variables among themselves, the Yo-Yo test results (distance travelled and VO_2_ max) correlate positively with the CMJ and Abalakov test (*p* = 0.001 in all cases).

## 4. Discussion

The present research provides new references on the performance variables in male beach handball players. This provides useful information, since it is important to measure the improvement of players with objective tests, to establish strategies both at the physical-sports and dietary levels, to improve the results, so this can translate into better results in competition. The participants were the best beach handball players in Spain. In summary, after observing the results, the initial hypothesis can be confirmed; the results in the performance variables point to differences between junior and senior players, however there are no significant differences between the playing positions. Furthermore, the results of these tests were also positively correlated with the adherence to the Mediterranean diet.

Firstly, focusing on body composition, in all basic measurements, there were differences between the junior and senior players. As shown in a recently published study [7], there are also differences in the variable muscle mass kg, fat mass kg and bone mass kg, and in the skinfolds (triceps, biceps, thigh, calf and the sum of the six skinfolds) and in the perimeters (arm, contracted arm and waist). In all cases the values are higher in the seniors, because of the different developmental stages of the junior versus senior players. However, there were no differences in the body composition and the anthropometric profile, according to the playing position.

Secondly, the performance tests carried out on the subjects and, in comparison with the study carried out by Martínez-Rodríguez et al. [6] on the women’s beach handball national teams (junior and senior), it can be seen that all of the results in the men’s categories were higher than those achieved by the women (Yo-Yo test; Men: junior 607.0 ± 214.0 m and senior 696.0 ± 194.0 m/Women: junior 382 ± 81.4 m and senior 414 ± 109 m; Abalakov jump; Men: junior 40.5 ± 6.14 cm and senior 44.6 ± 8.29 cm/Women: junior 28.5 ± 5.69 cm and senior 35.1 ± 6.89 cm; handgrip; Men: junior 46.6 ± 6.4 kg and senior 57.8 ± 12.01 kg/Women: junior 31.8 ± 3.37 kg and senior 35.1 ± 3.84 kg). Regarding the countermovement jump (CMJ), the results obtained here (junior 34.0 ± 5.31 cm and senior 37.8 ± 5.45 cm) were lower than those obtained by Martínez-Rodríguez et al. [5] (junior 43.7 ± 14.6 cm and senior 40.4 ± 13.6 cm) for the male category and also lower in comparison with the female senior category (39.6 ± 2.5 cm) and higher in the female junior category (30.1 ± 5.9 cm).

In relation to the Yo-Yo test, the data obtained here were superior (junior 607.0 ± 214.0 m and senior 696.0 ± 194.0 m), compared to those obtained by Martínez-Rodríguez et al. [6] (junior 382 ± 81.4 m and senior 414 ± 109 m) in the senior female category. The test selected was used because it has been demonstrated [39] that the Yo-Yo test specifically supports the handball achievements of elite players, meaning that this test, in addition to others, measures the important qualities for success in handball. However, this comparison should be taken with caution, as when comparing the results obtained in the present research with those of [39], significantly lower values were found; the main cause was the playing surface, since, in this study, the players performed the tests on sand.

An important finding of the present research, that coincides with other research [24], is the significant negative correlation between the BMI and CMJ. In other words, the players with the highest BMI were those who performed less countermovement jumping. There were also significant positive correlations between the variables of height, sitting height and wingspan with handgrip scores, so players with a greater overall height, trunk height and wingspan, had more upper limb strength. This has also been seen in professional female basketball players [40], Olympic pistol shooters [41] and volleyball players [42].

Coinciding with NBA basketball players [43] and handball players [44], the players who participated in the present research also presented wingspan values higher than height. It has been seen [44] that a greater wingspan implies a greater occupation of spaces in defensive and offensive actions, this aspect in sports, in which defensive movements using the arms and throws are involved, is a competitive advantage.

Another relevant finding is the significant negative correlations between the weight and the BMI with the results of the Yo-Yo test (both distances covered and VO_2_ max) and with the adherence to the Mediterranean diet. This means that heavier players have a worse aerobic endurance and also have a poorer dietary pattern.

As previously mentioned, the Mediterranean diet is well known for its health benefits, so it is of great importance that athletes, both professional and recreational, adhere to this dietary pattern [45]. The present investigation shows that professional handball players have an average adherence to the Mediterranean diet, both juniors and seniors. However, it should be noted that if the results of this research are compared with the results of women in the same category and sport [6], the results are slightly higher in the case of men.

If the results are compared with other similar studies in an athletic population, they do not always all go in the same direction. Sánchez-Benito et al. found that young female cyclists had a low or no adherence to the MD [46]. The same was true for elite indoor football athletes, where a low adherence to MD was observed [26].

The present study has certain limitations. First, its cross- sectional design avoids any causal conclusions. The KIDMED questionnaire was used to assess the players’ dietary quality, but not the quantity. It would be an interesting exercise to be able to make seven-day records to be able to evaluate both the quality and quantity.

## 5. Conclusions

The present cross-sectional study carried out with male players of the BH men’s national teams indicates that the anthropometric characteristics were superior in the senior players. A positive correlation was found between the adherence to the Mediterranean diet and the different performance test results. Those players who have a greater adherence to the Mediterranean diet, complete greater distances and obtain higher VO_2_ max values. Similarly, a positive correlation was found between the CMJ and weight, the CMJ and the BMI, and the same occurs in the correlation between the ABK and weight, and the ABK and the BMI; therefore, if the players have a lower weight and BMI, they will be able to reach a higher jump height. In addition, it has been possible to establish the conclusion that the greater the weight and BMI, the shorter the distance covered by the players in the Yo-Yo test, therefore the player will have a lower aerobic capacity and consequently a lower capacity to recover from the intermittent efforts (a fundamental aspect in the BH). It should also be considered that better results in the Yo-Yo test will improve the jumping ability (CMJ and Abalakov). The characteristics of the environment in which the BH takes place, sand, are a limitation for the present study.

## Figures and Tables

**Table 1:** Anthropometric characteristics.

	Total (*n* = 36)			Junior (*n* = 18)			Senior (*n* = 18)			*t* Test		
	Mean	SD	Range	Mean	SD	Range	Mean	SD	Range	*p*	MD	ES
Age (years)	20.9	5.55	(16–35)	16.7	0.46	(16,17)	25.0	5.19	(19–35)	<0.001	−8.28	−2.247
Height (cm)	184	7.81	(173–208)	181	5.90	(174–190)	188	7.73	(173–208)	0.002	2.291	−1.113
Seated height (cm)	96.4	3.73	(89.6–104)	94.1	3.17	(89.6–101)	98.6	2.79	(94.1–104)	<0.001	0.995	−1.514
Wingspan (cm)	189	9.46	(176–220)	184	7.45	(176–203)	193	9.35	(179–220)	0.004	2.817	−1.046
Weight (kg)	84.1	14.0	(63.5–115)	78.1	12.2	(63.5–98.3)	90.1	13.4	(67.8–115)	0.008	4.275	−0.940
BMI (kg/m^2^)	24.6	2.73	(19.8–30.4)	23.9	2.82	(19.8–28.4)	25.4	2.50	(21.7–30.4)	0.107	0.889	−0.552

*n* = number per group; SD = standard deviation; cm = centimetres; kg = kilograms; m = meters; BMI = body mass index; MD = mean differences; ES = effect size.

**Table 2:** Descriptive statistics and differences by category (junior vs. senior).

	Total (*n* = 36)			Junior (*n* = 18)			Senior (*n* = 18)			ANCOVA Comparison (Adjusting for BMI)		
	Mean	SD	Range	Mean	SD	Range	Mean	SD	Range	MD	*p*	ES
CMJ	35.8	5.64	(24.6–48.3)	34.0	5.31	(24.6–46.1)	37.8	5.45	(29.9–48.3)	−5.52	<0.001	−1.31
ABA	42.5	7.45	(32.0–63.4)	40.5	6.14	(32.0–57.4)	44.6	8.29	(33.2–63.4)	−6.36	0.003	−1.11
HG	52.2	11.1	(37.0–99.8)	46.6	6.42	(37.0–62.0)	57.8	12.0	(46.9–99.8)	−10.5	0.004	−1.08
YO-YO	650	207	(360–1160)	607	214	(360–1040)	696	194	(480–1160)	−157	0.007	−1.01
VO_2_ max.	41.9	1.74	(39.4–46.1)	41.5	1.79	(39.4–45.1)	42.3	1.63	(40.4–46.1)	−1.32	0.007	−1.01
KIDMED	6.78	2.38	(2–11)	6.83	2.26	(3–11)	6.72	2.56	(2–10)	−0.459	0.554	−0.207

*n* = number per group; SD = standard deviation; CMJ = countermovement jump; ABA = Abalakov test; HG = handgrip; VO_2_ max. = maximum oxygen quantity; KIDMED = Mediterranean diet quality index; MD = mean differences; ES = effect size.

**Table 3:** Differences, according to the playing position.

	Goalkeepers (*n* = 6)			Wings (*n* = 12)			Specialist (*n* = 6)			Pivots (*n* = 5)			Defenders (*n* = 7)			ANCOVA (Adjusted for the BMI)		
	Mean	SD	Range	Mean	SD	Range	Mean	SD	Range	Mean	SD	Range	Mean	SD	Range	F	*p*	ω^2^
BMI (kg/m^2^)	25.4	3.14	(19.8–28.4)	23.1	2.22	(20.2–27.5)	24.6	2.51	(21.5–28.0)	26.1	2.51	(23.7–30.4)	25.5	2.93	(21.2–28.3)			
Height (cm)	187	3.11	(184–192)	180	7.27	(173–192)	185	12.2	(174–208)	188	6.05	(182–195)	186	6.50	(174–194)	0.643	0.636	−0.039
Seated height (cm)	98.3	2.04	(95.1–101)	94.5	3.04	(89.6–101)	95.4	4.84	(89.7–104)	97.8	1.74	(95.6–100)	97.9	4.84	(90.0–104)	0.850	0.505	−0.015
Wingspan (cm)	190	6.22	(181–199)	184	7.88	(177–200)	193	16.7	(176–220)	193	6.82	(186–200)	189	6.00	(178–195)	0.709	0.592	−0.032
Weight (kg)	88.9	10.9	(71.7–102)	75.1	10.7	(63.5–95.4)	84.9	15.6	(64.8–109)	92.9	13.9	(79.8–115)	88.6	15.3	(69.9–107)	0.540	0.708	−0.015
CMJ	35.0	6.48	(24.6–41.0)	38.3	5.13	(31.5–48.3)	34.4	2.97	(31.0–38.9)	36.8	7.13	(28.3–46.6)	32.7	5.97	(29.8–46.1)	0.907	0.473	−0.009
ABA	41.6	6.01	(32.0–46.7)	44.6	6.72	(34.3–58.3)	40.5	4.81	(33.6–47.0)	45.1	11.7	(34.9–63.4)	39.5	8.22	(33.2–57.4)	0.951	0.449	−0.004
HG	50.2	7.93	(37.0–58.4)	49.1	10.1	(40.5–73.3)	51.0	4.50	(43.4–55.8)	64.5	20.4	(48.0–99.8)	51.7	5.44	(41.8–57.9)	1.620	0.195	0.065
YO-YO	552	203	(360–840)	683	209	(400–1160)	707	317	(400–1160)	616	66.9	(560–720)	640	183	(360–960)	0.678	0.613	−0.027
VO_2_ max.	41.0	1.70	(39.4–43.5)	42.1	1.75	(39.8–46.1)	42.3	2.66	(39.8–46.1)	41.6	0.562	(41.1–42.4)	41.8	1.54	(39.4–44.5)	0.678	0.613	−0.027
KIDMED	4.83	2.93	(2–10)	7.58	1.68	(5–10)	8.00	2.37	(5–11)	6.80	1.79	(5–9)	6.00	2.58	(3–10)	1.68	0.180	0.064

*n* = number per group; SD = standard deviation; BMI = body mass index; cm = centimetres; kg = kilograms; m = meters; CMJ = countermovement jump; ABA = Abalakov test; HG = handgrip; VO_2_ max. = maximum oxygen quantity; KIDMED = Mediterranean diet quality index; MD = mean differences; ω^2^ = partial omega squared.

**Table 4:** Correlations between the variables included in the study.

		Height	Seated Height	Wingspan	Weight	BMI	CMJ	ABA	HG	YO-YO	VO_2_ Max.	KIDMED
Height	Pearson’s r	—										
	*p* value	—										
Seated height	Pearson’s r	0.866 **	—									
	*p* value	<0.001	—									
Wingspan	Pearson’s r	0.899 **	0.730 **	—								
	*p* value	<0.001	<0.001	—								
Weight	Pearson’s r	0.793 **	0.749 **	0.702 **	—							
	*p* value	<0.001	<0.001	<0.001	—							
BMI	Pearson’s r	0.429 *	0.472 *	0.371 *	0.888 **	—						
	*p* value	0.009	0.004	0.026	<0.001	—						
CMJ	Pearson’s r	−0.182	−0.162	−0.084	−0.422 *	−0.491 *	—					
	*p* value	0.295	0.352	0.631	0.012	0.003	—					
ABA	Pearson’s r	−0.237	−0.210	−0.172	−0.471 *	−0.517 *	0.924 **	—				
	*p* value	0.171	0.227	0.322	0.004	0.001	<0.001	—				
HG	Pearson’s r	0.504 *	0.436 *	0.542 **	0.420 *	0.253	0.204	0.229	—			
	*p* value	0.002	0.008	<0.001	0.011	0.136	0.239	0.186	—			
YO-YO	Pearson’s r	−0.221	−0.206	−0.213	−0.492 *	−0.569 **	0.523 *	0.517 *	−0.019	—		
	*p* value	0.202	0.235	0.219	0.003	<0.001	0.001	0.001	0.912	—		
VO_2_ max.	Pearson’s r	−0.221	−0.206	−0.213	−0.492 *	−0.569 **	0.523 *	0.517 *	−0.019	1.000 **	—	
	*p* value	0.202	0.235	0.219	0.003	<0.001	0.001	0.001	0.912	<0.001	—	
KIDMED	Pearson’s r	−0.251	−0.193	−0.263	−0.406 *	−0.418 *	0.138	0.110	−0.168	0.512 *	0.512 *	—
	*p* value	0.140	0.259	0.122	0.014	0.011	0.429	0.528	0.329	0.002	0.002	—

BMI = body mass index; CMJ = countermovement jump; ABA = Abalakov test; HG = handgrip; VO_2_ max. = maximum oxygen quantity; KIDMED = Mediterranean diet quality index; * *p* value < 0.005; ** *p* value < 0.001.

## Data Availability

The data presented in this study is available upon request from the corresponding author. The data are not publicly available, due to the personal health information.
